# Therapy dose, recovery phase, and patient factors influencing language outcomes in post-stroke aphasia

**DOI:** 10.1016/j.clinsp.2026.101152

**Published:** 2026-10-01

**Authors:** Laura Carbonero Sancho, Olalla Saiz-Vázquez, Juan Hilario Ortiz-Huerta, Montserrat Santamaría-Vázquez

**Affiliations:** Universidad de Burgos, Health Sciences Department, España

**Keywords:** Aphasia, Stroke, Speech-language therapy, Therapy dose, Intensity, Language rehabilitation, Recovery phase

## Abstract

**Background:**

Therapy dose is increasingly recognized as an important factor influencing language recovery in post-stroke aphasia, yet evidence from routine clinical practice remains limited.

**Objective:**

To examine the relationship between total therapy dose and language outcomes, and to explore the influence of sex, recovery phase, and aphasia type.

**Methods:**

A longitudinal pre-post study was conducted with 20 adults with aphasia who received eight weeks of individualized speech-language therapy in three rehabilitation centers in Spain. Language performance was assessed with the Spanish version of the Mississippi Aphasia Screening Test (MASTsp). Statistical analyses included Wilcoxon tests, Spearman correlations, regression models, and non-parametric comparisons.

**Results:**

Participants showed significant improvement after intervention (p = 0.003). Therapy dose correlated positively with language gains (*r* = 0.624, p = 0.003), and receiving ≥ 20 h predicted greater improvement (p = 0.019). Sex and aphasia type did not significantly influence outcomes. Recovery phase showed a significant effect, with individuals in the acute and subacute stages improving more than those in the chronic phase (p = 0.046).

**Conclusions:**

A minimum of 20 therapy hours over eight weeks appears beneficial for language recovery in post-stroke aphasia. Recovery phase emerged as a relevant factor, while sex and aphasia type showed no measurable impact. These findings contribute practice-based evidence supporting the role of therapy dose in aphasia rehabilitation.

## Highlights

• At least 20 therapy hours over 8-weeks improved post-stroke aphasia.
• Therapy dose correlated strongly with language gains (*r* = 0.624).
• Acute and subacute patients improved more than chronic patients.
• Sex and aphasia type did not significantly affect therapy outcomes.
• Real-world evidence was obtained from Spanish rehabilitation centres.

## Background

The American Speech-Language-Hearing Association (ASHA)^1^ defines aphasia as an acquired neurological language disorder resulting from brain injury (most commonly due to stroke or traumatic brain injury). Aphasia affects up to one-third of stroke survivors and is among the sequelae with the greatest impact on autonomy, communicative functioning, and quality of life.^2^, ^3^, ^4^, ^5^ From the perspective of communicative functioning, aphasia can result in substantial limitations in activity and participation, as emphasized in the International Classification of Functioning, Disability and Health (ICF),^6^ underscoring the need for effective, evidence-based speech-language interventions.^7^^,^^8^

Across decades of clinical practice in speech-language pathology, diverse profiles of language impairment have been identified in people with aphasia (affecting phonological, lexical, semantic, syntactic, or pragmatic processing).^9^ This variability has driven the development of multiple diagnostic classification systems.^8^^,^^9^ One of the most influential contributions in this regard was the Boston classification system, developed in the 1960s by Norman Geschwind, Frank Benson, Harold Goodglass, and Edith Kaplan, and later formalized in the Boston Diagnostic Aphasia Examination by Goodglass and Kaplan in 1972,^8^ accompanying the Boston Diagnostic Aphasia Examination.^10^ This typology, which includes Broca’s aphasia, transcortical motor aphasia, global aphasia, mixed transcortical aphasia, Wernicke’s aphasia, transcortical sensory aphasia, conduction aphasia, and anomic aphasia, remains a key reference for clinical assessment, therapy planning, and research internationally.

With respect to language rehabilitation, the literature consistently highlights that the trajectory and severity of deficits are influenced by multiple factors (including lesion size and location, premorbid status, timeliness of medical care, and time to initiation of therapy).^11^ Early intervention is particularly critical, as the most substantial changes in language typically occur in the first weeks or months post-stroke. Nevertheless, approximately one-fifth of patients experience permanent language disability.^4^^,^^12^^,^^13^ This ongoing need highlights the importance of optimizing therapeutic parameters to maximize recovery outcomes.

In recent years, the concepts of therapeutic dose, frequency, and intensity have gained increasing relevance in speech-language pathology due to their relationship with principles of neuroplasticity and experience-dependent learning.^14^^,^^15^ Dose is typically understood as the total amount of therapy delivered, frequency as the number of hours or sessions per week, and intensity as the daily therapeutic load.^7^^,^^16^^,^^17^ Despite this growing interest, substantial methodological heterogeneity persists across studies, making it difficult to establish clear clinical recommendations regarding the optimal dose of language rehabilitation. This challenge is exacerbated in real-world clinical contexts, where resource availability, differences across healthcare systems, and individual patient needs result in wide variability in the amount of therapy delivered.^15^

Moreover, contemporary approaches to language rehabilitation increasingly emphasize personalized intervention models, recognizing that patient-specific variables (such as sex, aphasia type, and post-stroke phase) may modulate treatment response.^18^ However, most studies examining the effectiveness of speech-language therapy do not incorporate these factors simultaneously, limiting the clinical applicability of their findings.^19^^,^^20^

Therefore, the present study aims to explore the effect of speech-language therapy dose in individuals with post-stroke aphasia and to examine the moderating role of individual variables (sex, stroke phase, and aphasia type). By integrating these elements, this work seeks to contribute empirical evidence that supports the optimization of therapy planning and advances more effective and personalized intervention models in aphasia rehabilitation.

## Method

### *Study design*

A quasi-experimental longitudinal study with a single-group pretest–posttest design and no control group was conducted between January 2024 and May 2025 in three private neurological rehabilitation centres in Spain. This design allowed the examination of language performance changes following an eight-week speech-language therapy programme delivered under real clinical conditions.

### *Participants*

The sample was obtained through non-probabilistic convenience sampling. Twenty-two individuals were initially screened for eligibility, and, after verification of inclusion and exclusion criteria, twenty participants initiated and completed the eight-week intervention period, while two withdrew before the start of the study due to logistical difficulties. The inclusion criteria required a clinical diagnosis of post-stroke aphasia, an age between thirty and eighty years, a minimum pre-stroke educational level ensuring functional literacy, native Spanish as first language, absence of premorbid cognitive or physical impairment, and no prior exposure to neuromodulation treatments such as tDCS or rTMS. Individuals were excluded if they presented an unstable clinical condition or additional neurological disorders not related to stroke. Demographic and clinical characteristics were also collected, including type of stroke, lesion laterality, time post-stroke at enrolment and aphasia type. All participants provided written informed consent after receiving detailed information about the study. The protocol was approved by the Ethics Committee of the University of Burgos (code IO35/2025) and was conducted in accordance with the principles of the Declaration of Helsinki.

### *Measures*

Language performance was evaluated using the Spanish version of the Mississippi Aphasia Screening Test (MASTsp),^21^ a brief tool designed to detect and quantify impairments in spoken expression, auditory comprehension, reading and writing. The instrument consists of nine subtests and yields a maximum score of one hundred points, with higher scores indicating better performance. he original MAST was developed and initially validated by Nakase-Thompson and colleagues,^22^ and the Spanish adaptation has demonstrated strong psychometric properties, including high inter-rater reliability above 0.90 and convergent validity with other aphasia measures.^21^ The MASTsp was selected due to its brevity, sensitivity to change and feasibility for repeated administration in multicentre clinical studies.

### *Procedure*

The study began following approval from the ethics committee and the corresponding institutional registration. Recruitment then commenced across the participating centres. After eligibility was confirmed and informed consent obtained, baseline assessment was carried out using the MASTsp. Participants subsequently received an eight-week individualized speech-language therapy programme. Although the specific therapeutic procedures varied according to individual needs, all interventions were delivered by speech-language pathologists with more than five years of clinical experience in neurological communication disorders and were based on evidence-supported approaches, including semantic and phonological treatments, naming therapy, language stimulation techniques and activity- and participation-oriented strategies aligned with the ICF framework. The treatment dose was not predetermined by the research team, as the intervention took place in private settings where the number of weekly sessions depended on each participant’s economic circumstances, family availability and capacity to attend therapy. Nevertheless, the total number of therapy hours received during the intervention period was systematically recorded for each participant. At the end of the eight-week programme, the post-intervention assessment was administered under conditions equivalent to the baseline evaluation and by the same professionals. The overall flow of participant selection, inclusion, follow-up and analysis is presented in an adapted CONSORT-style diagram.

### *Data analysis*

Statistical analyses were conducted using IBM SPSS version 28.^23^ Given the small sample size and the non-normal distribution of the data assessed with the Shapiro-Wilk test, non-parametric methods were applied. Pre- to post-intervention changes were analyzed using the Wilcoxon signed-rank test. The association between total therapy dose and language improvement was examined through Spearman’s correlation, followed by a simple linear regression to determine whether dose predicted change in language performance. Differences in improvement according to sex, stroke phase (acute, subacute or chronic) and aphasia type were analyzed using the Mann-Whitney *U* and Kruskal-Wallis tests with a descriptive objective. Additionally, a moderation analysis following the procedure described by Hayes was performed to explore whether these variables acted as moderators of the relationship between treatment dose and language improvement. Finally, a multiple linear regression was used to examine the combined contribution of sex, stroke phase and aphasia type to the variability in treatment-related change. The significance level was set at α = 0.05. Given the small sample size, both the moderation analyses and the multiple linear regression were conducted solely for exploratory purposes, and should therefore be interpreted with caution in light of the increased risk of Type II error.

## Results

### *Sample characteristics*

The final sample consisted of twenty participants with post-stroke aphasia, seven men (35%) and thirteen women (65%). The mean age was 57-years (SD = 15), with a range from 29 to 75 years. As described in the Methods section, the chronic phase was defined as more than six months post-stroke. At baseline, 60% of the participants were in the chronic phase, 30% were in the acute phase and 10% in the subacute phase (Table 1).

**Table 1:** Descriptive statistics. Participant characteristics (n = 20).

Demographic characteristics	
Sex	
Male	7 (35%)
Female	13 (65%)
**Mean Age**	57.4 ± 15.4
**Educational level**	
Basic education	7 (35%)
Higher education	6 (30%)
University degree	3 (15%)
**Diagnosis**	
Left ischaemic stroke	12 (60%)
Left haemorrhagic stroke	6 (30%)
Right haemorrhagic stroke	1 (5%)
**Recovery phase**	
Acute phase	6 (30%)
Subacute phase	2 (10%)
Chronic phase	12 (60%)
**Type of aphasia**	
Global aphasia	2 (10%)
Mixed transcortical aphasia	2(10%)
Broca’s aphasia	2 (10%)
Transcortical motor aphasia	2 (10%)
Wernicke’s aphasia	1 (5%)
Transcortical sensory aphasia	4 (20%)
Conduction aphasia	4 (20%)
Anomic aphasia	3 (15%)

Regarding stroke etiology, the most frequent diagnosis was left ischaemic stroke (60%), followed by left haemorrhagic stroke (30%) and right haemorrhagic stroke (5%). The distribution of aphasia types included transcortical sensory aphasia (20%), conduction aphasia (20%), anomic aphasia (15%), global aphasia (10%), mixed transcortical aphasia (10%), Broca’s aphasia (10%), transcortical motor aphasia (10%) and Wernicke’s aphasia (5%). The mean MASTsp score at baseline was 68.00 (SD = 20.21) and 75.25 (SD = 19.86) at post-intervention. The Shapiro-Wilk test confirmed non-normality for the main variables, justifying the use of non-parametric analyses (Table 1).

### *Improvement in language performance after intervention*

To examine language change from pretest to posttest, the Wilcoxon signed-rank test was applied. Results indicated a significant improvement in MASTsp performance following intervention (*Z* = 2.940, p = 0.003). A Spearman correlation was conducted to analyse the association between total therapy dose and improvement in MASTsp scores. The results showed a positive and statistically significant correlation (*r* = 0.624, p = 0.003). Because therapy dose was also considered of clinical interest, an additional exploratory categorization was performed using a threshold of ≥ 20 versus < 20 h. This threshold was not derived post hoc from the present dataset, but was selected as a literature-informed reference point based on previous studies suggesting that greater language gains are often associated with total therapy doses in the range of approximately 20–50 hours.^7^^,^^14^ The simple linear regression analysis indicated that dose explained 24.2% of the variance in language improvement (F(1,17) = 6.73, p = 0.019, R = 0.533, R² = 0.284, adjusted R² = 0.242). Fig. 1 illustrates the association between total therapy dose and change in MASTsp score, showing a positive trend alongside substantial inter-individual variability.

**Fig. 1:**
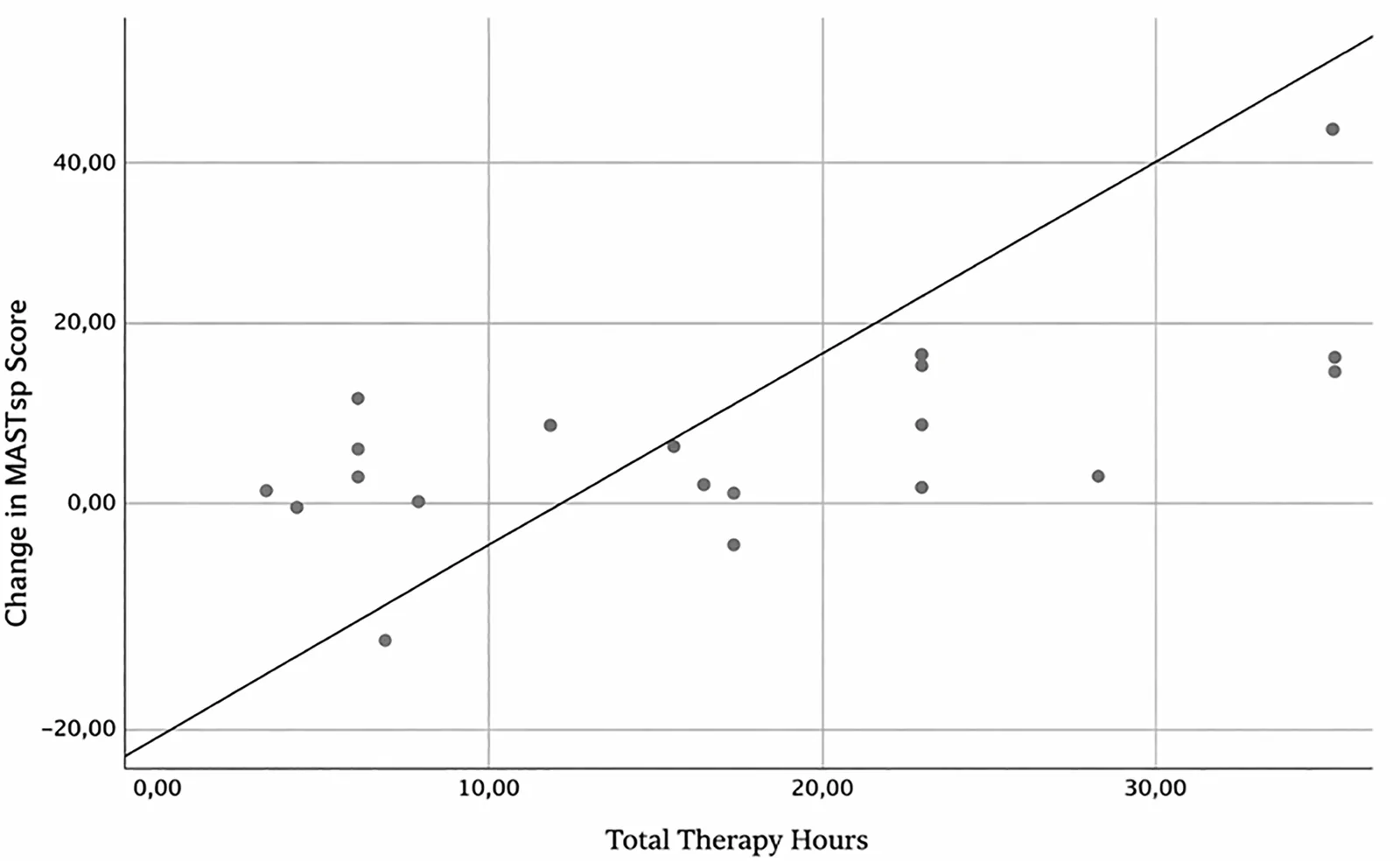
Scatterplot showing the relationship between total therapy dose (hours) and change in MASTsp score after the intervention period.

### *Improvement by sex, recovery phase and type of aphasia*

To determine whether improvements differed according to sex, recovery phase or aphasia type, Mann-Whitney U and Kruskal-Wallis tests were performed (descriptive values are presented in Table 2). No statistically significant differences in language improvement were detected for sex (*U* = 0.715, p = 0.485, *r* = 0.16) or aphasia type (*H* = 6.371, p = 0.497, η^2^ = −0.052). In the case of the analysis of the type of aphasia, and due to the groups having a small number of participants, this is a purely descriptive analysis that would need to be repeated in studies with a larger sample; therefore, this result should be interpreted with the utmost caution. However, significant differences were observed according to recovery phase (*H* = 6.156, p = 0.046, η^2^ = 0.24), indicating that time post-stroke influenced the magnitude of improvement.

**Table 2:** Differential MASTsp scores by sex, recovery phase and type of aphasia.

	Compared groups	Test statistic	p	Effect size
Sex	Male vs. Female	*U =* 0.715	0.485	*r =* 0.16
Recovery phase	Acute, Subacute, Chronic	*H =* 6.16	0.046^a^	η^2^ = 0.244
Type of aphasia	Global aphasia, Mixed transcortical aphasia, Broca’s aphasia, Transcortical motor aphasia, Wernicke’s aphasia, Transcortical sensory aphasia, Conduction aphasia, Anomic aphasia	*H =* 6.37	0.497	η^2^ = *−*0.052

MASTsp, Mississippi Aphasia Screening Test (Spanish version).

a p < 0.05.

### *Moderation by sex, recovery phase and type of aphasia*

A moderation analysis following Hayes’ (2013) procedure was conducted to assess whether sex, recovery phase or aphasia type acted as moderators of the relationship between therapy dose and language improvement. None of the variables showed a significant moderating effect: sex (β = −0.195, SE = 0.472, p = 0.685), recovery phase (β = 0.035, SE = 0.244, p = 0.888) or aphasia type (β = −0.213, SE = 0.130, p = 0.122). These results should be interpreted with caution.

### *Combined influence of sex, recovery phase and type of aphasia*

A multiple linear regression analysis was then carried out with the aim of determining whether sex, recovery phase and aphasia type jointly explained variance in language improvement. The model was statistically significant (*F*(3,16) = 4.667, p = 0.016, *R* = 0.683, R^2^ = 0.467, adjusted R^2^ = 0.367). The adjusted R^2^ indicates that, after accounting for the number of predictors included in the model, 36.7% of the variance in language improvement was explained, providing a more conservative and realistic estimate of model fit in a small sample.

## Discussion

This study analyzed the evolution of language performance in twenty individuals with post-stroke aphasia after eight weeks of speech and language therapy. The intervention was delivered by experienced Speech-Language Pathologists (SLPs) across three neurological rehabilitation centres in Spain and was assessed using the Spanish version of the Mississippi Aphasia Screening Test (MASTsp). In addition to language scores, sociodemographic and clinical variables and the total dose of therapy were collected, with the primary objective of examining whether the amount of intervention was associated with improvements in language performance.

Participants showed significant improvements after the intervention, a finding consistent with previous literature supporting the essential role of speech-language therapy in neural reorganization and language recovery following neurological injury.^24^^,^^25^ The improvement observed in this heterogeneous sample, treated in different clinical centres, suggests that speech-language therapy may be beneficial even in real-world clinical contexts with methodological variability, which increases the ecological validity of the study.

Correlation analyses revealed a positive association between therapy dose and improvement on the MASTsp: the greater the number of hours received, the greater the progress. This result aligns with meta-analyses and systematic reviews suggesting that higher therapeutic doses may promote deeper neuroplastic changes and improved functional outcomes.^3^^,^^7^^,^^17^^,^^26^ Likewise, the exploratory categorized analysis suggested that receiving at least 20-hours of therapy over eight weeks was associated with greater language improvement, which is broadly consistent with the 20–hour range proposed in previous research as a potentially relevant therapeutic window. However, this threshold should be understood as a literature-informed exploratory reference rather than as a definitive cut-point.

However, an important consideration is that most studies recommending these higher doses are based on specific intensive approaches such as Constraint-Induced Aphasia Therapy (CIAT) or Intensive Language Action Therapy (ILAT) (which require several hours of structured intervention per day for short periods).^24^^,^^25^ In contrast, the present study did not control for the specific therapeutic methods used in sessions (an aspect that represents a methodological limitation). At the same time, this variability reflects real clinical practice in Spanish rehabilitation centres, where no single standardized protocol exists, and treatment is tailored to each patient’s needs.

At the same time, this heterogeneity also represents an important limitation for the internal validity of the study. Participants differed in several clinically relevant variables, including time post-stroke, aphasia type, lesion aetiology and location, and therapy content across centres. These uncontrolled sources of variability may have influenced treatment response and therefore make it difficult to attribute the observed improvements specifically to therapy dose. Consequently, the relationship identified between therapy dose and language improvement should be interpreted as an association observed under real-world clinical conditions, rather than as evidence of a direct causal effect. As shown in Fig. 1, although a positive association was observed between therapy dose and language improvement, substantial variability remained across participants, likely reflecting the influence of uncontrolled clinical and therapeutic factors.

When examining individual variables, no statistically significant differences were detected in language improvement according to sex or aphasia type. However, these findings should be interpreted with caution, as the small sample size and the very limited number of participants within each aphasia subtype substantially reduced the power to detect potentially meaningful differences. By contrast, the finding for recovery phase appears more interpretable, as this comparison involved fewer groups and relatively greater statistical power, particularly in the acute and chronic phases. The absence of statistically significant findings for sex and aphasia type may reflect the limited statistical power of the study together with the marked clinical variability of the sample. The literature on the role of sex is inconsistent. For example, Brady et al.^16^ reported sex-dependent differences depending on therapy intensity (men responding better to high-intensity interventions and women to moderate intensity), while other studies have not found such distinctions.^27^^,^^28^

In contrast, recovery phase emerged as a relevant factor: individuals in the acute or subacute phases showed greater improvements than those in the chronic phase.^29^, ^30^, ^31^ This pattern is consistent with research indicating that the initial weeks and months after stroke represent a period of heightened neural plasticity.^5^ These results suggest that, although speech-language therapy is beneficial at any stage, early intervention may maximize recovery potential. In Europe, and particularly in Spain, where research on intensive therapy and recovery modulation remains limited, this finding adds preliminary evidence with potential clinical relevance.^32^^,^^33^

Although this study did not detect statistically significant effects related to aphasia type, previous research has noted that language difficulties are often analyzed by severity level rather than classical aphasia classification.^7^ Given that this study did not assess severity using specific tools and the subgroup numbers were small, no conclusions can be drawn in this regard.

The study presents several limitations. First, the small sample size (n = 20) substantially restricts the generalizability of the findings and limits the statistical power of the analyses, increasing the risk of Type II error, that is, failing to detect true associations between the variables examined. This issue is particularly relevant for analyses involving aphasia type, as the corresponding subgroups were very small (approximately 1–4 participants per category), which markedly reduces the ability to detect between-group differences and increases the likelihood that potentially meaningful effects remained unidentified. Accordingly, subgroup comparisons by aphasia type should be considered purely descriptive and interpreted with extreme caution. Likewise, the regression, moderation, and multiple-predictor models should be understood as exploratory, given the risk of overfitting associated with modelling several predictors in such a limited sample. Given the limited sample size, additional sensitivity analyses excluding outliers or specific subgroups were not performed, as they would likely have produced unstable estimates with limited interpretability. Second, it was not possible to control the type of intervention applied across centres, which, although reflective of real clinical practice, limits the ability to establish causal relationships between observed improvements and specific therapeutic strategies. Another limitation is the use of the MASTsp as the sole assessment tool. Although it is a brief instrument with adequate psychometric properties and is useful as a screening-level measure of core language abilities, it does not provide a comprehensive assessment of functional communication in everyday contexts or of the psychosocial impact of aphasia. As a result, relevant dimensions such as communicative participation, quality of life, and patient-perceived communication difficulties were not captured in the present study. Accordingly, the findings should be interpreted as reflecting changes in core language performance rather than broader functional or psychosocial outcomes. Despite the existence of widely used international instruments: Aachen Aphasia Test AAT^34^; Communication Activities of Daily Living, Second Edition CADL-2^35^; Communicative Effectiveness Index CETI^36^; Stroke and Aphasia Quality of Life Scale-39 SAQOL-39^37^ and Aphasic Depression Rating Scale ADRS,^38^ the lack of validated Spanish versions prevented their use in this study and may have limited the breadth of the findings. In addition, the marked heterogeneity of the sample and intervention conditions limited the internal validity of the study. Participants differed in recovery phase, aphasia type, lesion characteristics, and therapy content across centres, which introduce potential confounding factors and make it difficult to attribute observed improvements specifically to therapy dose. Therefore, the present results should be interpreted cautiously, and future studies with larger and more balanced samples are needed to clarify the impact of these variables on language recovery.

This study also presents several strengths that enhance its contribution to the field. It provides novel evidence in the Spanish and European context regarding the relationship between therapeutic dose and language recovery (an area that remains underexplored). Data collection across three centres with varied methodologies increases ecological validity and reflects real clinical practice. The concurrent analysis of sex, recovery phase, and aphasia type provides a broader exploratory perspective on factors potentially influencing language improvement. Moreover, the use of the MASTsp allowed systematic monitoring of change in core language performance without interfering with therapy.

In particular, this study represents one of the first approaches in Spain and Europe to analyzing therapy dose in real clinical environments (helping to expand a still-limited evidence base). It also identifies recovery phase as a key factor associated with language improvement (paving the way for future research exploring this effect in greater depth). Additionally, the findings encourage further investigation into the role of sex, comparisons across different therapeutic methodologies and intensity levels, and analysis not only of total dose but also of the quantity and nature of therapeutic inputs (elements that may support the design of more effective and individualized interventions).

## Conclusion

The findings of this study indicate a positive association between therapeutic dose and language recovery. Specifically, the analyses suggested that receiving at least 20-hours of speech-language therapy over an 8-week period was associated with greater improvement in core language performance. This threshold may be clinically relevant as a literature-informed exploratory reference, although the finding should be considered hypothesis-generating and requires confirmation in larger, preferably pre-registered studies. Additionally, the data suggest that recovery phase may influence clinical outcomes, with acute and subacute phases showing greater sensitivity to improvement compared to the chronic phase. No significant differential effects of sex or aphasia type were detected in this sample; however, these findings should be interpreted cautiously given the limited statistical power, particularly for analyses involving aphasia type. Overall, these conclusions should be interpreted with caution due to the small sample size and the heterogeneity of the sample and intervention conditions. Further research with larger and more homogeneous cohorts is needed to replicate these findings and strengthen the evidence regarding the optimal therapeutic dose and the factors that modulate language recovery after stroke.

## Ethics approval and consent to participate

The study was approved by the Ethics Committee of the University of Burgos (code IO35/2025). Written informed consent was obtained from all participants prior to enrolment.

## Consent for publication

All participants provided written consent for the publication of anonymized data.

## Use of artificial intelligence

The authors declare that no generative artificial intelligence (AI) tools were used in the writing, data analysis, or creation of figures for this manuscript. Only standard editing tools were used.

## Authors’ contributions

L.C.S. and O.S.V. conceptualized the study. L.C.S., J.H.O.H., and M.S.V. contributed to the methodology. Formal analysis and investigation were carried out by L.C.S. and O.S.V. J.H.O.H. and M.S.V. provided resources. Data curation, visualization, and the original draft were completed by L.C.S. All authors contributed to the review and editing of the manuscript. O.S.V. supervised and administered the project.

## Declaration of competing interest

The authors declare that there is no conflict of interest related to this study. The authors alone are responsible for the content and writing of this manuscript. This work did not receive funding from public agencies, commercial institutions, or nonprofit organizations.

## Data Availability

The datasets generated and analysed during the current study are available from the corresponding author on reasonable request.
